# Landscape of Consensus-Based Entity-Endorsed Perioperative Quality Measures

**DOI:** 10.1097/AS9.0000000000000559

**Published:** 2025-02-28

**Authors:** Alex H.S. Harris, Kristen Davis-Lopez, Eric Schmidt, Kenneth Nieser, Nader N. Massarweh

**Affiliations:** From the * Veterans Affairs Health Services Research and Development Center for Innovation to Implementation, Palo Alto Veterans Affairs Health Care System, Palo Alto, CA; † Department of Surgery, Stanford University, Palo Alto, CA; ‡ VHA Office of Performance Measurement, Analytics and Performance Integration, Quality and Patient Safety, Veterans Health Administration, Washington, DC; § Atlanta VA Health Care System, Surgical and Perioperative Care, Decatur, GA; ‖ Division of Surgical Oncology, Department of Surgery, Emory University School of Medicine, Atlanta, GA; ¶ Department of Surgery, Morehouse School of Medicine, Atlanta, GA.

**Keywords:** health care quality measures, performance measures

## Abstract

**Objective:**

Healthcare quality measures have a central role in monitoring and incentivizing the quality of surgical care. Payors and other stakeholders rely on consensus-based entities (CBEs) for the rigorous, independent evaluation and endorsement of quality measures. The aim of this study is to catalog current CBE-endorsed, surgery-related quality measures and to assess measure characteristics, gaps, and redundancies to inform prioritization of measure development efforts.

**Methods:**

The National Quality Forum Quality Positioning System and Battelle Partnership for Quality Measurement were reviewed to identify CBE-endorsed quality measures related to perioperative care. Identified measures were characterized in terms of their type (eg, structure, process and outcome), quality domain (eg, effectiveness and efficiency), focus (eg, complications, cost and improvement in functioning), and perioperative specialty (eg, general surgery and anesthesia).

**Results:**

A total of 172 perioperative measures were identified, of which 79 were currently CBE-endorsed and 93 were previously endorsed. Among currently endorsed measures, 43 (54%) were clinical outcomes measures (eg, mortality and/or complications, readmissions), 8 (10%) were patient-reported outcomes measures, 20 (26%) were process measures, 4 (5%) were cost measures, and 4 (5%) were measures of structure. Measures specific to cardiothoracic (n = 40) and orthopedic (n = 11) surgery were the most common while 12 measures were relevant to multiple specialties.

**Conclusions:**

Despite the large number of surgery-related quality measures, most are concentrated on a small sample of quality domains for a few surgical specialties. Fewer measures are focused on patient-reported outcomes or experiences, low-value care (eg, unnecessary testing or imaging), healthcare structures, or processes that may lead to better outcomes.

## INTRODUCTION

Surgery-related healthcare quality measures have a central role in monitoring the quality and safety of surgical care. These measures are also central to accountability programs for those who deliver services through compensation, reimbursement, and reputation.^1^ Yet, existing programs that collect data for and use quality measures are often resource intensive, their effectiveness for modifying clinical practice to improve outcomes is either modest or unknown, and can have unintended negative consequences.^2–5^ For example, the public reporting of quality data in the form of star ratings has been shown to both mitigate and exacerbate inequities in high-quality home health agency use.^6^ Thus, it is essential that the right metrics are captured using accurate methods and quality measure data are applied in ways that lead to a net benefit.^2^

Accordingly, the Medicare Improvements for Patients and Providers Act of 2008 requires the U.S. Department of Health and Human Services (HHS) to contract with a consensus-based entity (CBE) for the independent evaluation of quality measures before use in its programs. The Centers for Medicare and Medicaid Services (CMSs) and others rely on CBEs for the rigorous, independent evaluation and endorsement of quality measures. CBE-endorsed measures are central to many CMS programs such as the Hospital Value-Based Purchasing Program that adjusts payments to hospitals based on the quality of care they provide. Although CBEs have drawbacks, they provide some protection against the proliferation of measures that fail to meet evaluation criteria such as importance, validity, reliability, feasibility, usability, and harmonization with existing measures.^7^ In addition to various uses in CMS programs, CBE-endorsed measures are available for use in other institutions and contexts for accountability, benchmarking, and quality improvement initiatives.

Healthcare quality measures can generally be classified into the following types: structural measures, process measures, cost/resource use measures, outcome measures, or measures of patient experience.^8^ Distinct from these types of measures, taxonomies of quality domains have been developed and typically include safety, effectiveness, timeliness, efficiency, patient-centeredness, and equity.^9^ Measures of various types and domains are sometimes combined into composite measures. While it is possible there are measures of each type, in every domain, and for every surgical specialty, at present the current landscape of CBE-endorsed surgical quality measures has not been described.

Therefore, the primary purpose of this study is to review currently CBE-endorsed, surgery-related quality measures and to catalog existing measures by their characteristics relevant to evaluating quality and safety of surgical care. This will allow the identification of measurement gaps and redundancies and inform future measure development and retirement efforts. We hypothesize that coverage will be high in certain domains, but lacking in others.

## METHODS

We searched all quality measures in the repositories maintained by CMS’s former and current CBEs: the National Quality Forum Quality Positioning System^10^ and Battelle’s Partnership for Quality Measurement.^11^ Any organization, such as professional societies or healthcare systems, can seek review and endorsement for measures they develop through the current CMS CBE. Measures were included in the initial catalog if they directly measured aspects of surgical quality or the perioperative care of surgery patients. Abstracting and cataloging measures occurred September 2023 to May 2024. As defined in Table 1, we abstracted the attributes of each measure that might be of interest to measure developers, program evaluators, healthcare purchasers, and other stakeholders including measure definition and source, type of measure, National Academy of Medicine quality domain, measure focus, measure steward, perioperative specialty, and CBE endorsement status. We summarized the landscape of measure constructs by their attributes, presented as counts and percentages. The complete measure catalogue is available in the Supplemental Digital Content, see http://links.lww.com/AOSO/A481.

**TABLE 1.** Definitions of Extracted Attributes

Attribute	Definition/Values
Measure description and source	Measure name, description, and link to source material
Type	Clinical Outcome Measures: Physiologic states or health status as recorded in hospital record or data systems (mortality, complications, improvement in lab values or other tests). Composite Outcome Measure: Includes at least one clinical outcome measure bundled with other clinical outcome measure and/or process measures. Patent-Reported Outcome Measures: Data collected from patients on improvement in health status or experiences of care (improvements in functioning, satisfaction with treatment or treatment results). Process Measures: Signifies if patients in the target population receive specific healthcare processes, procedures, or encounters (appropriate antibiotic prophylaxis, timely follow-up care). Cost/Resource Use Measures: Quantifies the cost associated with specific episodes of care. Structural Measures:Captures infrastructure and other contextual factors (eg, participation in a QI registry) thought to be associated with better processes and outcomes.
IOM quality domain	Safe: Avoiding harm to patients from the care that is intended to help them. Effective: Providing services based on scientific knowledge to all who could benefit and refraining from providing services to those not likely to benefit (avoiding underuse and misuse, respectively). Patient-centered: Providing care that is respectful of and responsive to individual patient preferences, needs, and values and ensuring that patient values guide all clinical decisions. Timely: Reducing waits and sometimes harmful delays for both those who receive and those who give care. Efficient: Avoiding waste, including waste of equipment, supplies, ideas, and energy. Equitable: Providing care that does not vary in quality because of personal characteristics such as gender, ethnicity, geographic location, and socioeconomic status.
Measure focus	Characterizes the focus of the measure numerator (eg, mortality, cost)
Measure steward	The organization listed as the measure steward in the Partnership for Quality Measurement repository
Perioperative specialty	Perioperative specialty most aligned with the focus of the measure. If the measure denominator includes surgeries from multiple specialties, the measure is categorized as such.
CBE endorsement status	Endorsed vs. Reserve status: To be put on reserve status, measures must meet all of the CBE criteria except relating to an opportunity for improvement. Measures that were once but no longer endorsed are classified as “removed.”

## RESULTS

Of 172 individual measures identified, 79 were currently CBE-endorsed and 93 were once but not currently endorsed. Endorsement can be removed if a measure is not submitted for periodic maintenance review by the CBE or does not pass this review. Of the measures removed from endorsed status, 24 are Agency for Healthcare Research and Quality Patient Safety Indicators and other measures that are no longer submitted to the CMS-contracted CBE for maintenance review. Ten of the endorsed measures are on reserve status signifying they no longer have meaningful room for improvement.^12^ The attributes of the 79 CBE-endorsed measures are summarized in Table 2. Of the 43 (54%) clinical outcome measures and composites, 34 focused on short-term mortality and/or complications, 7 on readmissions, 2 on clinical markers of symptom improvement, while one of the outcome measures also included a process component (intervention response or documentation of a plan of care). Of the 8 (10%) patient-reported outcome measure (PROM) quality measures, 4 focused on improvement in functioning, 3 on the quality of shared decision-making from the patient’s perspective, and 1 on aspects of patient healthcare experience. Of the 20 (26%) process measures, 14 were medication-related (8 perioperative antibiotics; 6 other medications), 4 focused on procedural access/appropriateness, and 2 on timely follow-up. The 4 (5%) cost/resource use measures focused on the risk-adjusted cost to Medicare for patients receiving cataract surgery, knee arthroplasty, coronary artery bypass graft, and lumbar fusion. Of the 4 (5%) structural measures, 1 focused on surgical volume and 3 focused on participation in a clinical registry.

**TABLE 2.** Attributes of 79 CBE-Endorsed Perioperative Quality Measures

Type of Measure	N (%)	Measure Steward	N (%)
Clinical outcome Composite outcome Patient-reported outcome Process Cost/resource use Structure	35 (44) 8 (10) 8 (10) 20 (26) 4 (5) 4 (5)	The Society of Thoracic Surgeons Centers for Medicare & Medicaid Services Society for Vascular Surgery American Academy of Ophthalmology American College of Surgeons Massachusetts General Hospital American Heart Association Centers for Disease Control and Prevention Focus on Therapeutic Outcomes American Society of Anesthesiologist American Urogynecologic Society The Permanente Federation American College of Cardiology American Society of Plastic Surgeons Physician Consortium for Performance Improvement The Dartmouth Institute for Health Policy & Clinical Practice	33 (42) 20 (25) 6 (8) 3 (4) 2 (3) 2 (3) 2 (3) 2 (3) 2 (3) 1 (1) 1 (1) 1 (1) 1 (1) 1 (1) 1 (1) 1 (1)
IOM Quality Domain		Perioperative Specialty	
Safe Effective Efficient Patient-centered Timely Equitable	44 (56) 24 (30) 4 (5) 4 (5) 3 (4) 0 (0)	Cardiothoracic Multispecialty Orthopedic Vascular Ophthalmologic Breast General Transplant Urologic	40 (51) 12 (15) 11(14) 6 (8) 4 (5) 2 (3) 2 (3) 1 (1) 1 (1)
CBE Endorsement Status		Focu	
Endorsed Endorsed with reserve status	69 (87) 10 (13)	Mortality Surgical complications Mortality and complications Antibiotic administration Readmissions Other medication administration Improvement in clinical or patient-reported outcomes Appropriate procedure Cost Participation in a registry Shared decision making Appropriate follow-up Patient experiences Surgical volume	14 (18) 11 (14) 9 (11) 8 (10) 7 (9) 6 (8) 6 (8) 4 (5) 4 (5) 3 (4) 3 (4) 2 (3) 1 (1) 1 (1)

The most common National Academy of Medicine quality domain represented was safety (56%), primarily by measures of short-term mortality and surgical complications. The effectiveness domain (30%) was primarily represented by process measures and measures of patients’ response to treatment. Measures in the domains of efficiency (5%), patient-centeredness (5%), and timeliness (4%) were much less represented. No measures were related to equity.

The most common specialty-specific measures were related to cardiothoracic (n = 40) and orthopedic (n = 11) surgery. Seven of the orthopedic surgery measures were specifically related to elective knee and/or hip replacement. Twelve measures were not specialty specific (eg, perioperative antibiotic administration). Vascular, ophthalmologic, breast, general, transplant, and urologic surgery also had specialty-specific measures.

## DISCUSSION

Healthcare quality measures have become equally important and controversial in the contemporary healthcare landscape.^2,13^ The findings from this review suggest there are too many measures overall without enough measures that meet rigorous evaluation criteria (eg, feasibility, validity) and that sufficiently cover important domains of quality and patient safety. However, simply filling in the measurement gaps is not the answer. Rather, these data should be used to inform discussions about how to prioritize measurement development where additional evaluations data might benefit surgical practice and program implementation, prioritize measure harmonization to avoid redundancies where they exist, and prioritize measure retirement when quality and safety improvement efforts might best be focused elsewhere.

In contrast to measure scans in other clinical contexts,^14^ clinical outcome measures and composites were the most common—mostly focused on short-term mortality, complications, and readmissions. Measures of improvements in clinical or patient-reported outcomes are rarer and more limited to a few subspecialities and procedures. Collecting data for PROM-based quality measures is expensive and time-consuming for healthcare systems and patients. If the current efforts to use PROM-based quality measures in the context of elective knee and hip replacement are found to be logistically feasible and effective in driving improvements in care, then perhaps the use of these measures should be expanded to other procedures where case mix adjusted improvements in PROMs is expected to be high. However, the expense and effort to implement PROM-based quality measures, or any new measures for that matter, need to be justified by careful evaluations of their effectiveness.

Most existing measures are focused on safety and effectiveness. Far fewer measures target aspects of efficiency such as the provision of low-value care, for example, unnecessary preoperative testing for low-risk patients and procedures.^15^ Measures of patient-centeredness, timeliness, and equity are not well-represented. Measuring each of these domains involves logistical and measurement challenges,^3^ but without measures, we cannot assess quality in these domains.

Cardiothoracic surgery, specifically the Society for Thoracic Surgeons, has enthusiastically embraced quality measurement and management initiatives.^16^ But even these measures are narrowly focused on safety, and to a lesser degree effectiveness, rather than other domains of quality such as patient-centeredness, efficiency, or equity. In orthopedics, quality measures for the safety and effectiveness of elective hip and knee replacement are well developed and justified by the frequency and cost of these procedures. However, other areas of orthopedic procedures and subspecialties have low measure coverage. Multispecialty measures have appeal because they are not restricted to specific procedures; however, the question remains if the focus of these measures (eg, antibiotic prescribing) targets the ideal opportunities for improvement.

One of the major goals of this study was to provide an organized list of currently endorsed surgery-related quality measures, sortable by various measurement characteristics. However, a significant limitation of this study is that it will soon be out of date. The National Quality Measures Clearinghouse was a national resource for over 17 years^12^ that should be revived to produce a comprehensive and dynamic system of curation. It is also worth noting that the developers of many measures that were once CBE-endorsed no longer pursue maintenance review and endorsement, most prominently the Agency for Healthcare Research and Quality. Without independent and transparent review of these measures, it is difficult to know which of the previously endorsed measures should still be trusted for large-scale quality improvement or accountability programs. Also, many measures exist and are used by private and public institutions (eg, Leapfrog, Veterans Health Administration) that have never been evaluated by an independent CBE. We did not include these measures because they have not been independently reviewed and are of unknown quality. Nevertheless, our analyses of the current landscape of CBE-endorsed measures can inform thinking about which new measures might be complementary for monitoring and improving the quality of surgical care.

## Supplementary Material


